# Novel Medicines and Strategies in Cancer Treatment and Prevention

**DOI:** 10.1155/2014/474078

**Published:** 2014-05-27

**Authors:** Chih-Hsin Tang, Gautam Sethi, Po-Lin Kuo

**Affiliations:** ^1^Graduate Institute of Basic Medical Science, China Medical University, Taichung 404, Taiwan; ^2^School of Medicine, China Medical University, Taichung 404, Taiwan; ^3^Department of Biotechnology, College of Health Science, Asia University, Taichung 413, Taiwan; ^4^Department of Pharmacology, Yong Loo Lin School of Medicine, National University of Singapore, Singapore 119077; ^5^Graduate Institute of Clinical Medicine, Kaohsiung Medical University, Kaohsiung 807, Taiwan

Cancer is one of the leading causes of death around the world [1]. There are a number of challenges that the limitation of new drugs to potentiate antitumor therapies [2]. Furthermore, despite a positive response to initial treatment, many patients eventually develop a local recurrence and spread of the primary tumor [3–5]. Additional prognostic biomarkers are urgently needed to improve decision making regarding adjuvant therapy for these patients.

In response to the call for papers, we received many submissions from all over the world. After an initial screening, we selected eight articles that are proper in this special issue. All manuscripts underwent a very rigorous peer-review process. The full papers in this issue can be broadly organized into three main categories: (i) new anticancer agents, (ii) new antimetastasis agents, and (iii) new strategies in cancer prevention.

*(i) New Anticancer Agents*. Y. Ishima et al. have prepared* S-*nitrosated human serum albumin (SNO-HSA) with many conjugated SNO groups (poly-SNO-HSA) using chemical modification with 2-iminothiolane, which can be a reliable and safe NO donor. The poly-SNO-HSA can be used as an effective multifunctional antitumor agent because NO release from Poly-SNO-HAS is able to inhibit tumor cell growth and induce apoptosis “*Poly-s-nitrosated albumin as a safe and effective multifunctional antitumor agent: characterization, biochemistry and possible future therapeutic applications.*”

C-Y. Tu et al. found that histone deacetylases (HDACs) inhibitor, trichostatin A (TSA), is able to attenuate EGFR expression through induction of microRNA-7 expression, which is an off-target activity of TSA to improve the anticancer efficiency of lapatinib-based therapy via HDAC-independent manner “*Trichostatin A suppresses EGFR expression through induction of microRNA-7 in an HDAC-independent manner in lapatinib-treated cells*.”

*(ii) New Antimetastasis Agents*. Clinical reports have indicated that HER2 is frequently overexpressed in HBV-encoded X protein (HBx)-expressing HCC patients and is associated with their poor prognosis. In consistence to these findings, C.-M. Hung et al. showed that HBx is able to upregulate HER2 expression via HuR-dependent mRNA stabilization in HCC cells, which subsequently rendered HCC cells more metastatic. Therefore, in addition to targeting to HER2, RNA-binding protein, HuR, is as a new target for therapy in those patients “*Hepatitis B virus X upregulates HuR protein level to stabilize HER2 expression in hepatocellular carcinoma cells.*”

P.-C. Chen et al. describe the important roles of CCN family proteins in skeletal development, and abnormal expression of CCN proteins is related to the tumorigenesis of primary bone tumors such as osteosarcoma, Ewing sarcoma, and chondrosarcoma. CCN proteins could therefore serve as potential therapeutic targets for drug development against primary and metastatic bone tumors “*The CCN family proteins: modulators of bone development and novel targets in bone-associated tumors*.”

J.-C. Chen et al. also discussed various mechanisms and mediators that regulate the expression of integrins and integrin-mediated signaling, contributing to increased cell migration. Therefore, the development of new drugs that can selectively target regulators of integrin gene expression and ligand-integrin signaling might hold great promise for the treatment of chondrosarcomas “*Novel strategies for the treatment of chondrosarcomas: targeting integrins.*”

In addition, J.-Y. Kan et al. reported that even antimicrobial drug, gemifloxacin, could be a novel anticancer agent for the treatment of metastasis in colon cancer, which suppresses the activation of NF-*κ*B, leading to a decrease in snail expression “*Gemifloxacin, a fluoroquinolone antimicrobial drug, inhibits migration and invasion of human colon cancer cells.”*

*(iii) New Strategies in Cancer Prevention*. H. Orita et al. performed retrospective analysis of the connection between pfetin expression, clinical-pathological data and incidences of recurrence in the patients with gastrointestinal stromal tumor (GIST) that is the most common mesenchymal tumor of the digestive tract. The results demonstrate that lack of pfetin expression is an additional predictor of recurrence in resected GIST.

M. Wang et al. first studied the mutations of JAK2 V617F, FLT3-ITD, NPM1, and DNMT3A in Chinese patients with myeloproliferative neoplasms (MPN). The results indicated that patients bearing different forms of mutations may contribute to the pathogenesis.

All together, we hope this special issue will provide new inputs for those who are interested in the development of novel cancer prevention and treatment strategies.

*Chih-Hsin Tang* *Gautam Sethi* *Po-Lin Kuo*

## References

[1] Siegel R, Ma J, Zou Z, Jemal A (2014). Cancer statistics, 2014. *CA: A Cancer Journal for Clinicians*.

[2] Phelps MA, Sparreboom A (2014). A snapshot of challenges and solutions in cancer drug development and therapy. *Clinical Pharmacology & Therapeutics*.

[3] Michor F, Nowak MA, Iwasa Y (2006). Evolution of resistance to cancer therapy. *Current Pharmaceutical Design*.

[4] Quail DF, Joyce JA (2013). Microenvironmental regulation of tumor progression and metastasis. *Nature Medicine*.

[5] Rebucci M, Michiels C (2013). Molecular aspects of cancer cell resistance to chemotherapy. *Biochemical Pharmacology*.

